# Predictors of antiretroviral therapy initiation in eThekwini (Durban), South Africa: Findings from a prospective cohort study

**DOI:** 10.1371/journal.pone.0246744

**Published:** 2021-02-19

**Authors:** Karla Therese L. Sy, Shema Tariq, Gita Ramjee, Kelly Blanchard, Cheng-Shiun Leu, Elizabeth A. Kelvin, Theresa M. Exner, Anisha D. Gandhi, Naomi Lince-Deroche, Joanne E. Mantell, Lucia F. O’Sullivan, Susie Hoffman

**Affiliations:** 1 Department of Epidemiology, Boston University School of Public Health, Boston, MA, United States of America; 2 Institute for Global Health, University College London, London, United Kingdom; 3 South African Medical Research Council, HIV Prevention Research Unit, Durban, South Africa; 4 Ibis Reproductive Health, Cambridge, Massachusetts and Johannesburg, South Africa; 5 Division of Gender, Sexuality, and Health, HIV Center for Clinical and Behavioral Studies, New York State Psychiatric Institute and Columbia University, New York, New York, United States of America; 6 Department of Biostatistics, Mailman School of Public Health, Columbia University, New York, New York, United States of America; 7 Department of Epidemiology & Biostatistics, CUNY Graduate School of Public Health and Health Policy, City University of New York, New York, New York, United States of America; 8 CUNY Institute for Implementation Science in Population Health, City University of New York, New York, New York, United States of America; 9 Department of Psychology, University of New Brunswick, Fredericton, Canada; 10 Department of Epidemiology, Mailman School of Public Health, Columbia University, New York, New York, United States of America; Hofstra University, UNITED STATES

## Abstract

Despite expanded antiretroviral therapy (ART) eligibility in South Africa, many people diagnosed with HIV do not initiate ART promptly, yet understanding of the reasons is limited. Using data from an 8-month prospective cohort interview study of women and men newly-diagnosed with HIV in three public-sector primary care clinics in the eThekwini (Durban) region, South Africa, 2010–2014, we examined if theoretically-relevant social-structural, social-cognitive, psychosocial, and health status indicators were associated with time to ART initiation. Of 459 diagnosed, 350 returned to the clinic for their CD4+ test results (linkage); 153 (33.3%) were ART-eligible according to treatment criteria at the time; 115 (75.2% of those eligible) initiated ART (median = 12.86 weeks [95% CI: 9.75, 15.97] after linkage). In adjusted Cox proportional hazard models, internalized stigma was associated with a 65% decrease in the rate of ART initiation (Adjusted hazard ratio [AHR] 0.35, 95% CI: 0.19–0.80) during the period less than four weeks after linkage to care, but not four or more weeks after linkage to care, suggesting that stigma-reduction interventions implemented shortly after diagnosis may accelerate ART uptake. As reported by others, older age was associated with more rapid ART initiation (AHR for 1-year age increase: 1.04, 95% CI: 1.01–1.07) and higher CD4+ cell count (≥300μL vs. <150μL) was associated with a lower rate of initiation (AHR 0.38, 95% CI: 0.19–0.80). Several other factors that were assessed prior to diagnosis, including stronger belief in traditional medicine, higher endorsement of stigma toward people living with HIV, food insecurity, and higher psychological distress, were found to be in the expected direction of association with ART initiation, but confidence intervals were wide and could not exclude a null finding.

## Introduction

In response to compelling evidence demonstrating that timely antiretroviral therapy (ART) initiation substantially decreases morbidity and mortality in people living with HIV (PLHIV) [1–3] and also prevents onward transmission, World Health Organization (WHO) guidelines were revised in 2015 to recommend expanding ART eligibility to all individuals regardless of CD4+ count or WHO clinical stage. UNAIDS also set new HIV diagnosis and treatment targets, with the goal that by 2020, 90% of all PLHIV will know their HIV status, 90% of those diagnosed with HIV will receive sustained antiretroviral therapy, and 90% of those on ART will have viral suppression. In 2016, South Africa formally adopted these new WHO guidelines [4] and implemented a strategy of immediate ART initiation regardless of CD4+ count [5]. Recent research has demonstrated that previously-implemented expansions in ART eligibility criteria promoted timely ART initiation. For example, a 2018 multi-country analysis demonstrated that the incidence of ART initiation within 6 months increased after ART eligibility expansions between 2007 and 2015, particularly in younger adults [6]. Another study, in the rural subdistrict of Hlabisa in KwaZulu-Natal, the same South African province where our study was conducted, found that expansion of ART eligibility resulted in an 85% increase in ART initiation among newly-eligible patients and a 32% increase in the number of patients starting ART overall [7].

However, even with ART eligibility expansion and improved access to treatment in South Africa, persistent barriers remain in optimizing scale-up of HIV care and reaching near-to-universal ART initiation. Research conducted prior to universal eligibility has shown that there is considerable loss in the HIV care continuum before ART initiation even among people who were eligible under the guidelines prevailing at that time [8–11]. In 2017, only 68.8% of people who knew they were PLHIV were on ART in South Africa [12]. Moreover, a 2018 study in rural KwaZulu-Natal, South Africa, demonstrated that only 60% initiated ART within the study period (August 2011 to December 2012), and the number on treatment decreased with increasing CD4+ count [13]. A community-randomized trial of Universal Test and Treat conducted from 2013 to 2018 demonstrated that communities receiving universal ART plus combination prevention did not have greater ART coverage relative to communities receiving ART according to local guidelines (with or without combination prevention) [14]. To meet the UNAIDS 90-90-90 targets, further research needs to assess the health-seeking behaviours of people who test HIV-positive to identify what factors prompt or deter them from initiating ART.

Previous work in South Africa has primarily focused on time to ART initiation associated with clinical, sociodemographic, household, and health system factors [15–17]; however, there is much less research examining the role of a range of social-structural, social-cognitive, and psychosocial factors on ART initiation. Some insight can be found in a 2018 systematic review of the qualitative literature, which cited the influence of sociodemographic and socioeconomic factors, health status, stigma, and social-cognitive factors such as HIV beliefs and knowledge on patient decision-making about ART initiation [18], but quantitative evidence is limited. In the present study, conducted among a cohort of newly-diagnosed PLHIV, we considered those who were ART-eligible according to the respective criteria in effect at the time they linked to care, and examined factors that might promote or hinder subsequent ART uptake. Although the data used in these analyses were collected prior to the policy of immediate ART initiation, our findings are relevant to current efforts to enhance time to ART initiation because the data set included a wide range of potentially important variables that often are not available.

## Materials and methods

### Study design

Data were drawn from *Pathways to Engagement in HIV Care for Newly-Diagnosed South Africans*, a prospective cohort study conducted between 2010–2014. Participants were recruited from among those who sought HIV counselling and testing (HCT) at three public sector primary healthcare clinics (PHC) in the eThekwini region of KwaZulu-Natal [19], the South African province with the highest reported HIV prevalence at 17.4% [20]. Two of the public sector clinics included were located in urban areas and one site was in a rural area. The clinics provided primary healthcare services and ART. The main aim of the parent study was to examine factors associated with more rapid or delayed linkage to care, defined as returning to the clinic after diagnosis to obtain CD4+ test results [19]. For the current analysis, we focused on the subset of individuals who both linked to care and were eligible for ART. We defined ART eligibility according to the South Africa National Guidelines during the time of each participant’s linkage to care (until April 2010: CD4+ count ≤200 cells/μL or WHO stage IV; April 2010 to August 2011: CD4+ count ≤350 cells/μl for people with tuberculosis and pregnant women; August 2011 to January 2015: all adults with CD4+ count ≤350 cells/μl) [21]. Because these public sector clinics did not at this time create individual medical records until the patient initiated ART, we relied on careful interviewing methods to ascertain dates of ART initiation and CD4+ count.

### Participants

The enrolled cohort comprised 459 women and men who were newlydiagnosed with HIV; 350 participants (76%) linked to care during the study; of these, 32 did not report CD4+ count values and were excluded from the analysis, and 153 (43.7%) were eligible for ART based on the criteria above.

Recruitment methods, assessment, and cohort follow-up have been described in detail elsewhere [19,22]. Briefly, individuals presenting for HIV testing at one of the three clinics were interviewed by study staff not affiliated with the clinic before and after standard HCT. Among those otherwise eligible for the study (age 18 or over, not planning to relocate within 1 year), newly diagnosed HIV-positive, and willing to enroll, a structured baseline interview was conducted within 30 days of HIV diagnosis. Follow-up interviews were conducted at 4- and 8-months post-diagnosis regardless of whether the participant had returned to the clinic for subsequent care. The median follow-up time in the study cohort was 39.7 weeks; median time to linkage to care–defined as self-report of completing CD4+ count testing and returning to the clinic to obtain results–was 10.71 weeks [19].

All procedures were approved by the Institutional Review Board of the NYS Psychiatric Institute/Columbia University Department of Psychiatry and the Biomedical Research Ethics Committee of the University of KwaZulu-Natal. Written informed consent was obtained prior to the screening interview, and an additional written informed consent was obtained prior to the baseline interview for the cohort study.

### Measures

#### Outcome

The main outcome of this analysis was ART initiation, defined as a self-report that the participant had started taking antiretrovirals (ARVs). To measure this variable, participants were asked at each of the three assessments if they had made any medical visits (excluding hospitalizations) since the previous interview, and the date, location, and reason for each visit (e.g., for ART initiation, CD4+ test results, pick up ARVs, etc.). Later in the interview, individuals were asked directly if they had initiated ART and, if so, when they initiated.

Based on the self-reported reasons for visits and the response to the direct question, we categorized each individual as having initiated ART (yes/no) if they reported any medical visits for ART initiation or if they responded that they were currently taking ARVs. Time to ART initiation was calculated as the number of days from linkage to care to the reported date of ART uptake and was converted to fractional weeks for ease of interpretation.

#### Exposures

As described previously [19], the conceptual framework for *Pathways to Care* posited that retention in the HIV care continuum for ART initiation is influenced by social-structural, social-cognitive, and psychosocial factors. We also wanted to determine whether indicators of HIV disease severity were associated with ART initiation. Measures that had been developed, validated, and tested in sub-Saharan African settings were utilized when available. Moreover, we drew from our own elicitation work conducted prior to the study to develop valid items for several scales, such as barriers to care and expected positive and negative outcomes of enrolling in care (S1 Table).

Measures were examined in the following domains:

1. Social-structural factors were characterized as markers of societal-level inequalities or social processes that can influence individuals’ care-seeking behaviors; in this study they were measured at the individual level. These included age, gender, relationship status, and socioeconomic status (education, employment, and food insecurity [23], which were assessed during the screening interview. Although they are typically characterized as “sociodemographic factors,” we conceptualized the variables as individual-level markers of social-structural factors, given that gender, age, relationship status, and socioeconomic status represent socially-determined relationships that position individuals to have more or fewer resources, autonomy, and access to care, which can strongly influence subsequent engagement in care. Stigma measures, including HIV-related blame [24] and HIV-related anticipated shame [25], were assessed prior to diagnosis, and internalized and anticipated stigma [26] were assessed at the baseline interview. Gender-related barriers to seeking care, such as having household- or job-related responsibilities or having a partner who did not want the individual to attend the clinic, were assessed at the interview following HCT; living away from home for work or school was assessed at baseline; and other structural barriers to care such as distance to clinic (assessed via GPS based on information provided at the baseline and follow-up interviews), and travel time and cost to get to the clinic were elicited at screening.
2. Social-cognitive factors were drawn from the Information, Motivation, and Behavior (IMB) model [27], and included CD4 knowledge and treatment beliefs such as belief in positive and negative outcomes of enrolling in care (assessed during screening); beliefs about ART for HIV treatment and traditional medicine beliefs [28] (assessed during baseline); and satisfaction with care (assessed at the interview—baseline, 4-month or 8-month follow-up—at which linkage to care was reported).
3. Psychosocial measures included psychological distress (Kessler-10) [29,30] (assessed during screening), coping strategies [31] (baseline), and disclosure of status (assessed during baseline).
4. Health status indicators included CD4+ count, which was self-reported at the interview during which linkage to care was reported (prior to ART initiation); and report of any clinical criteria for WHO stage 3/4 disease (severe weight loss, unexplained chronic diarrhea, unexplained persistent fever, tuberculosis, etc.), which was self-reported at screening, prior to diagnosis [32].

The full description of measures used to assess each construct, source, inter-item consistency and reliability of the scales, how each variable was modeled, and timing of all assessments is presented in S1 Table.

### Statistical analysis

Descriptive statistics were generated to characterize the sample and Kaplan-Meier curves were employed to estimate the time to ART initiation among all participants. The primary analysis examined the association between the time to ART initiation and each exposure using Cox proportional hazards models. We also examined this association separately among men and women. Individuals were censored at their last interview date if they were lost to follow-up due to death, declining to continue with the study, or inability of the study team to locate them after multiple attempts. They were censored at their second follow-up assessment if they had not undergone ART initiation. We report the estimated hazard ratios (HR) and their corresponding 95% confidence interval (CI) as a measure of the strength of association. The proportional hazards assumption was checked among all crude and adjusted Cox proportional hazards models. The proportional hazards assumption was only violated for internalized stigma. Thus, we assessed the HRs of ART initiation and internalized stigma at two time periods where the proportional hazards assumption held: less than 4 weeks after linkage to care and greater than 4 weeks after linkage to care.

Initial models were adjusted only for clinic of recruitment. To adjust for the effect of other potentially confounding variables, a “minimally sufficient adjustment set,” based on a directed acyclic graph (DAG), was identified for each hypothesized causal factor (S1 Table). DAGs represent the hypothesized causal structure among variables and help to identify the appropriate set of variables to adjust for, called the “minimally sufficient adjustment set”. Adjusting for these variables allows for the estimation of the total average causal effect of each predictor while appropriately adjusting for confounding [33].

All analyses, except the proportional hazard assumption tests, were conducted in SPSS version 24.0.0.1 [34]. The proportional hazard assumption tests were conducted in SAS 9.4 [35].

## Results

**Fig 1:**
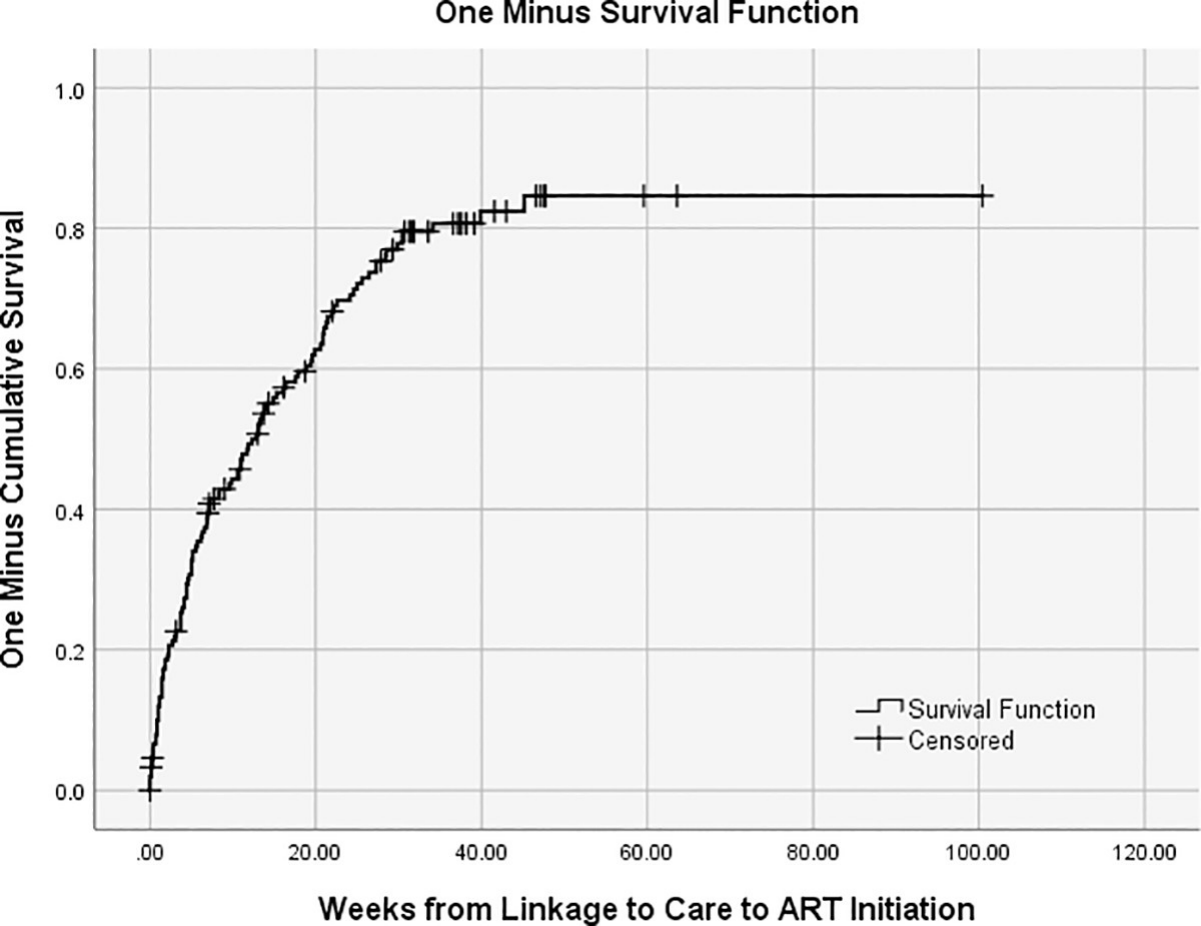
Time (weeks) from linkage to care to ART initiation.

Among the 153 individuals eligible for ART and therefore included in this analysis, the median age was 31 years (Interquartile range (IQR): 25–35), 65% were women, and half had a highest educational attainment of 9^th^ to 11^th^ grade (Table 1). Sixty-nine percent were unemployed and 43% reported at least some food insecurity. The median distance from home to the clinic by road was 2.5 km (IQR: 1–7), and the median CD4+ cell count at enrollment in care was 191 cells/μL (IQR: 119–254). There were 115 participants (75.2%) who reported initiating ART. The median time from linkage to care to ART initiation estimated from the Kaplan-Meier curve was 12.86 weeks (95% CI: 9.75, 15.97) (Fig 1). Table 1 also reports the demographic and testing characteristics of those who initiated and did not initiate ART. There were significant differences in the CD4+ count, age categories, and number of WHO stage 3/4 clinical criteria among those who initiated and did not initiate.

**Table 1:** Demographic and testing characteristics of 153 women and men living with HIV diagnosed at three primary healthcare clinics in the eThekwini, SA region, and eligible for ART at linkage to care.

	Whole Sample		No ART Initiation		ART Initiation	
	N	Median (IQR)	N	Median (IQR)	N	Median (IQR)
Age	153	31.0 (28.0–35.0)	38	29.0 (24.8–34.3)	115	31.0 (28.0–35.0)
Distance from home to clinic, km	149	2.5 (1.0–7.0)	37	3 (1.1–7.0)	112	2.5 (0.9–2.5)
CD4+ count (cells/μL)	153	191 (119–254)	**38**	**250 (194–284)**	**115**	**166 (106–246)**
	**N**	**%**	**N**	**%**	**N**	**%**
**Age**						
< 25	19	12%	9	**24%**	10	**9%**
25–29	43	28%	12	**32%**	31	**27%**
30–34	52	34%	8	**21%**	44	**38%**
> = 37	39	25%	9	**24%**	30	**26%**
**Gender**						
Women	99	65%	22	58%	77	67%
Men	54	35%	16	42%	38	33%
**Relationship Status**						
Not married or in a relationship	13	9%	2	5%	11	10%
Married/in a relationship, not living together	97	63%	24	63%	73	63%
Married/in a relationship, living together	43	28%	12	32%	31	27%
**Education**						
8th grade or less	32	21%	11	29%	21	18%
9th - 11th grade	82	54%	19	50%	63	55%
Grade 12- Matriculated	23	15%	6	16%	17	15%
Matriculation plus certificate, diploma, or additional degree	16	10%	2	5%	14	12%
**Employed**						
No	106	69%	26	68%	80	70%
Yes	47	31%	12	32%	35	30%
**Food Insecurity**						
Often	20	13%	4	11%	16	14%
Sometimes	36	24%	12	32%	24	21%
Seldom	10	7%	5	13%	5	4%
Never	87	57%	17	45%	70	61%
**Travel time to clinic**						
Less than half an hour	88	58%	15	39%	73	63%
Between half an hour and 1 hour	44	29%	7	18%	37	32%
More than 1 hour	6	4%	1	3%	5	4%
Unknown	15	10%	15	61%	0	0%
**Number of WHO stage 3/4 clinical criteria**^a^						
None	72	47%	12	**32%**	60	**52%**
One or more	81	53%	26	**68%**	55	**48%**
**CD4+ count (cells/**μ**L)**						
<150	52	34%	7	**18%**	45	**39%**
150 to 299	84	55%	23	**61%**	61	**53%**
> = 300	17	11%	8	**21%**	9	**8%**

Significant p-values (p<0.05) are highlighted in bold based on Mann-Whitney U test or Pearson chi-squared test.

In the clinic and confounder-adjusted hazard ratios (AHR) estimated from the Cox proportional hazard models (Table 2), each additional year of age was associated with a 4% higher rate of ART initiation (AHR 1.04, 95% CI: 1.01–1.07). The rate of ART initiation among those with CD4+ count equal to or above 300 cells/μL was 62% lower compared with those whose CD4+ count was less than 150 cells/μL (AHR 0.38, 95% CI: 0.19–0.80). However, having one or more WHO stage 3/4 clinical criteria (relative to none) corresponded with a 38% decreased rate of ART initiation (AHR 0.62, 95% CI: 0.41–0.92).

**Table 2:** Hazard ratios (HR) for hypothesized predictors of time to ART initiation (clinic-adjusted and adjusted for confounders) (N = 153).

			ART Initiation			
			Adjusted for clinic only		Adjusted for clinic and other confounders^a-k^	
	n	% of N	HR	95% CI	HR	95% CI
**Social structural factors**						
Age (continuous)^a^			**1.04**	**1.01–1.07**	**1.04**	**1.01–1.07**
Gender, men^a^ (ref: women)	54	35%	1.14	0.77–1.70	1.14	0.77–1.70
Education^c^ (ref: 8th grade or less)						
9th - 11th grade	82	54%	1.30	0.78–2.18	1.54	0.90–2.65
Grade 12- matriculated	23	15%	1.22	0.64–2.33	1.39	0.71–2.73
Matriculation plus certificate, diploma, degree	16	10%	1.39	0.70–2.74	1.61	0.80–3.24
Employed, yes^c^ (ref: no)	47	31%	1.09	0.72–1.64	1.08	0.70–1.65
Food Insecurity, ever^c^ (ref: never)	66	43%	0.84	0.58–1.24	0.75	0.51–1.12
Relationship status^b^ (ref: not married or in a relationship)						
Married/in a relationship, not living together	97	63%	0.89	0.47–1.68	1.24	0.63–2.45
Married/in a relationship, living together	43	28%	0.75	0.38–1.51	0.84	0.42–1.71
Away from home, yes^b^ (ref: no)	138	90%	1.40	0.72–2.73	1.26	0.64–2.46
Gender-related barriers^b^ (ref: 0)						
0.1 to 1	23	15%	1.06	0.63–1.77	1.17	0.69–2.00
1.1 to 2	16	11%	1.49	0.85–2.60	1.51	0.86–2.64
Internalized stigma, any, less than 4 weeks after linkage to care ^b^ (ref: none)	5	12%	**0.33**	**0.13–0.85**	**0.35**	**0.14–0.91**
Internalized stigma, any, greater than or equal to 4 weeks after linkage to care^b^ (ref: none)	39	35%	0.83	0.50–1.36	0.85	0.51–1.42
Anticipated stigma, high^b^ (ref: low)	28	18%	0.82	0.51–1.33	0.81	0.50–1.31
Shame, high^b^ (ref: low)	120	79%	0.82	0.52–1.29	0.85	0.55–1.34
Blame, high^b^ (ref: low)	116	76%	0.76	0.49–1.19	0.73	0.46–1.15
Travel time to clinic^d^ (ref: < ½ hour)						
1/2 hr.- 1 hr	44	32%	1.01	0.67–1.52	0.99	0.65–1.50
> 1 hr.	6	4%	0.85	0.34–2.13	0.88	0.34–2.23
GPS distance^d^ (ref: < 1.42 km)						
1.421–2.620 km	43	29%	0.80	0.50–1.28	0.80	0.50–1.30
> 2.62 km	56	38%	1.06	0.67–1.67	1.13	0.70–1.82
Cost for visit^d^ (ref: none)						
Between R1.00-R15.00	65	49%	1.07	0.70–1.63	1.10	0.72–1.68
R15.00+	16	12%	0.62	0.32–1.19	0.63	0.32–1.22
**Social-cognitive factors**						
CD4 knowledge^f^ (ref: none correct)						
1 correct	26	17%	1.16	0.59–2.26	1.05	0.53–2.07
2 correct	103	67%	1.19	0.68–2.09	1.11	0.63–1.95
Negative outcome beliefs, any^e^ (ref: none)	63	41%	1.11	0.76–1.62	1.04	0.70–1.55
Positive outcome beliefs, any^e^ (ref: none)	132	86%	1.13	0.66–1.95	1.31	0.75–2.30
ARV positive attitudes^e^ (ref: 1^st^ tertile)						
2nd tertile	40	26%	1.35	0.85–2.13	1.47	0.89–2.44
3rd tertile	48	31%	1.03	0.66–1.62	0.97	0.61–1.54
Stronger beliefs in traditional medicine^e^ (ref: 1^st^ tertile)						
2nd tertile	50	33%	1.34	0.86–2.11	1.22	0.75–1.99
3rd tertile	48	32%	0.70	0.43–1.12	0.66	0.40–1.07
Care satisfaction^k^ (ref: 1^st^ tertile)						
2nd tertile	36	24%	0.93	0.58–1.49	0.89	0.55–1.43
3rd tertile	57	38%	0.96	0.63–1.48	0.89	0.56–1.42
**Psychosocial factors**						
Psychological distress (Kessler) sum, elevated ≥ 16 ^h^ (ref: not elevated < 16)	12	8%	0.48	0.22–1.03	0.55	0.24–1.23
Disclosed by baseline interview, yes^j^ (ref: no)	102	67%	0.89	0.60–1.32	0.93	0.61–1.40
Coping acceptance^i^ (continuous)			0.84	0.70–1.01	0.86	0.72–1.04
Coping alcohol^i^ (continuous)			1.12	0.66–1.91	0.95	0.54–1.68
Coping positive reframing^i^ (continuous)			**0.77**	**0.60–0.98**	0.81	0.64–1.04
Coping denial^i^ (continuous)			1.12	0.78–1.61	1.07	0.73–1.58
Coping religion^i^ (continuous)			1.00	0.82–1.22	1.01	0.82–1.24
**Health status indicators**						
CD4+ count^g^ (ref: <150 cells/μL)						
150–299 cells/μL	84	55%	0.76	0.51–1.12	0.76	0.51–1.14
> = 300 cells/μL	17	11%	**0.37**	**0.18–0.76**	**0.38**	**0.19–0.80**
WHO stage 3/4 clinical criteria, one or more^g^ (ref: none)	81	53%	**0.66**	**0.46–0.96**	**0.62**	**0.41–0.92**

^a-k^As indicated below, variables included in adjusted models are based on the Directed acyclic graph (S1 Table).

^a^Adjusted for clinic.

^b^Adjusted for clinic, age, gender.

^c^Adjusted for clinic, age, gender, relationship status.

^d^Adjusted for clinic, education.

^e^Adjusted for clinic, age, gender, psychological distress, CD4 knowledge, education.

^f^Adjusted for clinic, age, gender, education.

^g^Adjusted for clinic, age, gender, delayed testing, education.

^h^Adjusted for clinic, age, gender, internalized stigma.

^i^Adjusted for clinic, age, gender, psychological distress.

^j^Adjusted for clinic, age, gender, psychological distress, gender barrier scale, anticipated stigma, ARV positive attitudes.

^k^Adjusted for clinic, CD4 knowledge, traditional medicine attitudes.

Significant p-values (p<0.05) are highlighted in bold.

Internalized stigma (relative to no internalized stigma) was associated with a 65% decrease in the rate of ART initiation (AHR 0.35, 95% CI: 0.19–0.80) during the period less than four weeks after linkage to care. However, internalized stigma was not associated with ART initiation four or more weeks after linkage to care.

Although none of the other factors hypothesized to influence rate of ART initiation were found to be statistically significant, several were associated with ART initiation in the direction we would expect. Endorsing strong (vs. weak) traditional medicine beliefs was associated with a 34% lower rate of ART initiation (AHR 0.66, 95% CI: 0.40–1.07). Higher level of education, particularly high school matriculation (vs. 8^th^ grade or less) was associated with a higher rate of ART initiation (AHR 1.61, 95% CI: 0.80–3.24). High level of psychological distress was associated with lower rate of ART initiation (AHR 0.55, 95% CI: 0.24–1.23), as were food insecurity (AHR 0.75, 95% CI: 0.51–1.12), higher visit cost (AHR 0.63, 95% CI: 0.32–1.22), and high levels of blame (AHR 0.73, 95% CI: 0.46–1.15). However, two other non-significant associations were in the opposite direction of what would be expected; scoring higher on positive reframing and acceptance as coping strategies were associated with a lower rate of ART initiation (Table 2). In the analyses stratified by gender, the associations between exposures and time to ART initiation did not suggest any important differences between men and women (S2 Table), except for age: younger women had lower rates of ART initiation relative to older women, but this difference was not observed among men.

In post-hoc analyses, we sought to understand why the two measures of disease severity (CD4+ count as a marker of immunosuppression, number of WHO stage 3/4 clinical criteria) were associated with ART initiation in opposite directions. The degree of immunosuppression and number of WHO stage 3/4 clinical criteria were not correlated (r = 0.002, p = 0.984). Of those with CD4+ counts less than 150 cells/μL, greater than half (27/52, 52%) did not report any WHO clinical criteria. Additionally, 43% (36/84) and 53% (9/17) of those with 150–299 cells/μL and 300 CD4+ cells/μL, respectively, did not report any WHO clinical criteria.

## Discussion

In this prospective study of PLHIV in South Africa who were newly linked to care, we found that experiencing internalized stigma shortly after enrolling in HIV care was associated with lower rate of ART initiation, yet internalized stigma after this early period did not alter ART initiation rate. We also observed a decrease, although nonsignificant, in the rate of ART initiation associated with endorsement of blame, another measure of stigma. These findings are consistent with a growing body of quantitative and qualitative research documenting that HIV-related stigma diminishes uptake of HIV testing, ART initiation, and ART adherence in South Africa [36–38], as well as more generally in the sub-Saharan context [18,39–45]. For example, Earnshaw and others also found an effect of internalized stigma assessed shortly after diagnosis on ART initiation, which in their study was mediated through avoidant coping [36]. Our findings demonstrate that HIV-related stigma hindered treatment initiation even in the context of increasing availability of ART and expansion of ART eligibility [44]. The results reinforce the need for stigma-reduction interventions to be incorporated into HIV counselling and treatment education, particularly during or immediately after linkage to care, as well as for continuing efforts to reduce HIV-related stigma at the facility and community levels [18,36].

We also found, as have others, that younger-aged individuals had lower rates of ART initiation [46]. Earlier analyses from this study demonstrated that younger individuals also took longer to initially link to care [19]. Younger populations in South Africa are currently disproportionally affected by HIV; recent data show that the number of adolescents seeking HIV care has notably increased in the past decade [47], and the number of adolescents aged 10–19 living with HIV has increased by 20% [48]. Stratified by gender, our results demonstrated that the association between younger age and longer time to ART initiation was driven by women, as no difference by age was observed among men. South African adolescent girls and young women remain extraordinarily vulnerable to acquiring HIV [48–51]. The results of this study suggest that disparities in access to treatment also exist for young women, and that special efforts are needed to support them to access care and treatment.

Consistent with previous work, PLWH in this study who had a greater degree of immunosuppression (i.e., lower CD4+ count) had a higher rate of ART initiation [13,15,52,53]. However, in our data CD4+ count and WHO stage 3/4 clinical indicators were not correlated, and those who reported at least one WHO clinical indicator (i.e., were in poorer health) had a lower rate of achieving ART initiation than those who reported no such indicators. To our knowledge, this finding has not been previously demonstrated and requires further investigation. Those with serious disease according to the WHO clinical criteria may have delayed ART because of stigma related to symptomatic HIV disease or because the conditions themselves prevented individuals from engaging in care. Another potential reason is that opportunistic infections associated with WHO stage 3/4 disease such as tuberculosis and cryptococcus required deferral of ART initiation.

Stronger belief in traditional medicine was also related to a lower, albeit non-significant, rate of ART initiation. The impact of traditional healers and medicine beliefs on ART initiation has not been extensively explored, but prior research has suggested a strong negative relationship between traditional, complementary, and alternative medicine use and ART adherence in sub-Saharan settings [54–57]. Our findings suggest that ART uptake may be influenced by similar factors.

This study had many strengths. In a clinic-recruited population, we measured psychosocial and social-cognitive exposures early in the HIV care cascade. The first interview was conducted prior to HIV-testing, allowing us to assess definitive temporality between exposures of interest and the outcome, and retention in the study was high. Measures were based on multi-item scales with good reliability and, when available, were developed and validated in sub-Saharan African settings. Further, utilizing a DAG added to the validity of our findings. With assessment of multiple exposures, the DAG allowed us to appropriately account for the confounders relevant to each exposure, rather than including every variable of interest in one model.

An important limitation of this study was that we relied on self-report of clinic visit dates and CD4+ count results without verifying them with medical record data. Public-sector primary health care clinics in KwaZulu-Natal created individual patient charts only after the individual initiated ART. Although we recruited patients who were newly-diagnosed in the clinics, the study staff did not have routine access to medical records to verify self-reported measures if and when ART was initiated. This meant that those who did not know or remember their CD4+ count (32 out of 350 participants) were excluded from the final analytic sample. However, it is unlikely that not knowing one’s CD4+ count would be associated with both the exposures and outcome, and thus any selection would not induce bias in the measures of association. We sought to reduce socially-desirable reporting by conducting interviews in a facility other than the clinic and by staff unaffiliated with clinic staff. We additionally attempted to limit misclassification of the outcome, ART initiation date, by asking about it in several different ways throughout the study interview. Any potential errors would likely be non-differential, which would bias associations towards the null. Misclassification of CD4+ count would likely also be non-differential—and therefore bias associations toward the null—because participants had no incentive to report a higher or lower value to study staff. Further, In our sample, only two people who linked to care were not successfully followed and they were censored in the analysis; thus, any bias due to loss to follow-up was limited. Finally, the small sample size in our study may have resulted in insufficient statistical power to detect some important associations.

The expansion of ART eligibility is a monumental step forward in reaching UNAIDS 90-90-90 treatment goals of nearly universal ART initiation in South Africa. However, beyond ART eligibility expansion, additional efforts are needed to ensure that all PLHIV receive care with minimal delay. Even though this study was completed prior to roll-out of universal ART-eligibility in South Africa and individual behavior may be different in the universal ART eligibility context, our findings remain highly relevant given that even under universal eligibility substantial numbers of PLHIV fail to seek ART care or get lost in the HIV care cascade prior to ART initiation.

## Conclusions

Our work adds to the body of evidence that internalized stigma, younger age, and higher CD4+ count influence HIV care continuum outcomes, particularly the timely initiation of ART. Multilevel interventions to reduce stigma and thereby promote earlier initiation of ART are urgently needed. Furthermore, efforts should focus on reconceptualizing strategies for the promotion of early ART to youth, as well as those with higher CD4+ count. It is essential to ensure that newly-diagnosed PLHIV return promptly for treatment to reach nearly universal ART initiation in South Africa and control the HIV epidemic.

## Supporting information

S1 Fig Directed acyclic graph for predictors of ART initiation^a^. ^a^ Each direct arrow represents an *a priori* hypothesized causal relationship between two variables. Age, Gender–adjusted for only clinic. Relationship status, Away from home. Gender barriers, Stigma—adjusted for clinic, age, gender. SES—adjusted for clinic, age, gender, relationship status. Visit distance/transport time/cost—adjusted for clinic, education. Treatment beliefs—adjusted for clinic, age, gender, psychological distress, CD4 knowledge, education. CD4 knowledge—adjusted for clinic, age, gender, education. CD4+ count/WHO stage clinical criteria—adjusted for clinic, age, gender, delayed testing, education. Distress—adjusted for clinic, age, gender, internalized stigma. Coping strategies—adjusted for clinic, age, gender, psychological distress. Disclosure—adjusted for clinic, age, gender, psychological distress, gender barrier scale, anticipated stigma, ARV positive attitudes. Care satisfaction—adjusted for clinic, CD4 knowledge, traditional medicine attitudes.
(TIF)

S1 Table Description of exposures. (DOCX)

S2 Table Hazard ratios (HR) for hypothesized predictors of time to ART initiation (adjusted for clinic and confounders) (N = 153) stratified by gender. (DOCX)
