# Supplementary figures and images for: Predictors of antiretroviral therapy initiation in eThekwini (Durban), South Africa: Findings from a prospective cohort study
Source: PLoS One. 2021 Feb 19;16(2):e0246744. doi: 10.1371/journal.pone.0246744 (PMC7895397; doi:10.1371/journal.pone.0246744)

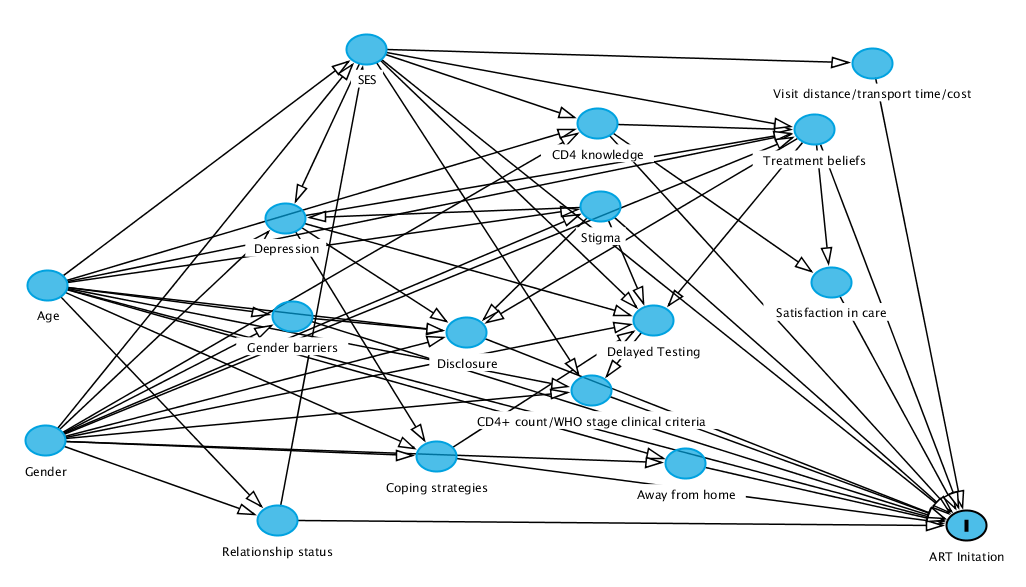

Supplement: S1 Fig — a Each direct arrow represents an a priori hypothesized causal relationship between two variables. Age, Gender–adjusted for only clinic. Relationship status, Away from home. Gender barriers, Stigma—adjusted for clinic, age, gender. SES—adjusted for clinic, age, gender, relationship status. Visit distance/transport time/cost—adjusted for clinic, education. Treatment beliefs—adjusted for clinic, age, gender, psychological distress, CD4 knowledge, education. CD4 knowledge—adjusted for clinic, age, gender, education. CD4+ count/WHO stage clinical criteria—adjusted for clinic, age, gender, delayed testing, education. Distress—adjusted for clinic, age, gender, internalized stigma. Coping strategies—adjusted for clinic, age, gender, psychological distress. Disclosure—adjusted for clinic, age, gender, psychological distress, gender barrier scale, anticipated stigma, ARV positive attitudes. Care satisfaction—adjusted for clinic, CD4 knowledge, traditional medicine attitudes. (TIF) [file pone.0246744.s001.tif]
