# Supplementary material for: Predictors of antiretroviral therapy initiation in eThekwini (Durban), South Africa: Findings from a prospective cohort study
Source: PLoS One. 2021 Feb 19;16(2):e0246744. doi: 10.1371/journal.pone.0246744 (PMC7895397; doi:10.1371/journal.pone.0246744)
Supplement: S1 Table — (DOCX) [file pone.0246744.s002.docx]

**S1 Table. Description of exposures**

| **Directed acyclic graph nodes** | **Construct and measures** | **Timing of assess-**  **ment**^a^ | **N of items** | **N of response options** | **How assessed; (sample) item/s** | **Cronbach’s alpha** | **Source** | **Coding in analysis** |
| --- | --- | --- | --- | --- | --- | --- | --- | --- |
| **Social structural factors** | | | | | | | | |
| Age | Age | Screening |  |  | Self-reported | N/A |  | Continuous variable |
| Gender | Gender of respondent | Screening | 1 |  | Observed by interviewer | N/A |  | Dichotomous variable - male, female |
| Socioeconomic Status (SES) | Education | Screening | **2** |  | *What is the highest grade in school that you completed? (by category); Do you have a certificate, diploma, or degree?* | N/A |  | 4-level categorical variable- <8^th^ grade; 9th-11^th^ grade; 12^th^ grade/matriculated; training or degree beyond matriculation |
|  | Employment | Screening | 1 |  | *How would you describe your employment situation?* | N/A |  | Dichotomous variable - Employed (part-time, full-time, occasional, self) vs unemployed (looking for work, not looking for work, unable to work, retired) |
|  | Food insecurity | Screening | 1 |  | *Do the people in your household go without food often, sometimes, seldom, or never?* | N/A | Jewkes, J., Stepping stones study(21) | Dichotomous variable - Never vs. ever (seldom, sometimes, often) |
| Relationship status | Relationship and living status | Screening | 2 |  | *Are you married (consensual or legal marriage)? Is there someone you have a relationship with and who you call your partner? Do you currently live with your ((husband/wife) OR regular partner)?* | N/A |  | 3-level categorical - Not married or in a relationship, Married/in a relationship, not living together,  Married/in a relationship, living together |
| Away from home | Away from home for work or school for > 1 month | Baseline |  |  | Participants were asked to list all they places might stay or call home, to indicate for how long they usually stayed there and for what reasons. | N/A | Developed for this study | Dichotomous - Currently working or studying and stays away from home for work or school for more than 1 month (Yes/No) |
| Gender barrier | Gender-related barriers to care | Post-test | 3 | 3 – *No, maybe, yes* | *Could [….] keep you from returning to the clinic in 2 weeks to get your CD4 results: Your partner does not want you to go to the clinic; having to take care of children or other family members; not being able to take time from work?* | 0.767 | Subset of barriers to care items, developed during formative work | 3-level categorical variable -  0, 0.1 to 1, 1.1 to 2 |
| Stigma | HIV-related blame | Screening | 2 | 4-*strongly disagree to strongly agree* | *People who have HIV/AIDS are cursed; …… must have done something wrong and deserve to be punished* | 0.746 | Adapted from Kalichman, et al., 2005(49) | Mean modeled as a dichotomous variable -<2 and >=2 |
|  | HIV-related anticipated shame | Screening | 2 | 4-*strongly disagree to strongly agree* | *I would be ashamed if I were infected with HIV/AIDS; …….if someone in my family was infected with HIV/AIDS* | 0.807 | Adapted from Genberg, et al., 2008(25) | Mean modeled as a dichotomous variable -<2 and >=2 |
|  | Internalized stigma | Baseline | 5 | 4-*never to most of the time* | *You have felt that you did not deserve to live; You have felt that you are no longer a person* | 0.852 | Negative Self-Perception sub-scale, HASI-P, Holzemer, et al., 2007 (22) | Mean modeled as a dichotomous variable - any vs. none |
|  | Anticipated stigma | Baseline | 5 | 4-*strongly disagree to strongly agree* | *If people know you are HIV+….*  *you will be abused by strangers; you will get support from people who are important to you* | 0.885 | Developed during formative work, and drawing from Discrimination Experiences subscale, HASI-P, Holzemer, et al., 2007 (22) | Mean modeled as a dichotomous variable - upper 20% vs. lower 80% |
| Logistical barriers (Distance/transport time/cost) | Travel time to clinic | Screening | 1 | 3 | *How long did it take you to travel to the clinic today? (by category)* | N/A |  | 3-level categorical variable- less than ½ hour; from ½ to 1 hour; more than 1 hour |
|  | Distance (km) by roads from home to clinic | Baseline |  |  | GPS used to calculate distance by roads from clinic (where tested or where they received CD4+ results, if elsewhere) to self-identified landmark near the participant’s home, plus additional distance from home to landmark determined from estimated walk-time to the landmark. | N/A |  | 3-level categorical variable - based on the distribution in the sample, <= 1.42 km, 1.421-2.62, > 2.62 |
|  | Cost | Baseline |  |  | *How much did it cost you to get there? (by category)* |  |  | 3-level categorical variable – None, between R1.00-R15.00, greater than R15.00 |
| **Social-cognitive factors** | | | | | | | | |
| CD4 knowledge | CD4 knowledge | Screening | 2 | *True, False, Not sure* | *A CD4 count measures how healthy you are; You have to have a CD4 test before you can get antiretroviral drugs from the clinic.* | N/A | Developed during formative work | Count of number correct, modeled as 3-level categorical variable– None, 1 correct, 2 correct |
| Treatment beliefs | Negative beliefs about outcomes of enrolling in care | Screening | 3 of 5 nega-tive items | *No, possibly, yes* | *If you return to the clinic for your CD4 results, do you think this will happen? The clinic staff will gossip about you in the community; You won’t learn anything at the clinic that will help you)* | 0.832 | Developed during formative work; factor analysis yielded 2 constructs | Mean modeled as a dichotomous (none/any) variable |
|  | Positive beliefs about outcomes of enrolling in care | Screening | 4 items | *No, possibly, yes* | *If you return to the clinic for your CD4 results, do you think this will happen?*  *The clinic staff will teach you how to stay healthy and strong;* *You will be on your way to getting care that will help you feel better.* | 0.374 -deemed too low for a scale | Developed during formative work; factor analysis yielded 2 constructs | Mean modeled as a dichotomous (none/any) variable |
|  | ARV attitudes | Baseline | 9 | 4-*strongly disagree to strongly agree* | *ARVs help most HIV+ people feel stronger; ARVs are not safe for HIV+ people.* | 0.855 | Developed during formative work | Mean modeled as a 3-level categorical (tertiles) variable |
|  | Traditional medicine attitudes | Baseline | 5 | 4-*strongly disagree to strongly agree* | *Umuthi has the power to make people better when they are ill; There are certain illnesses that only a traditional healer knows how to cure; Treatment from a traditional healer is not effective in controlling the HIV virus.* | 0.700 | Based on Liddell, 2008(27) | Mean modeled as a 3-level categorical (tertiles) variable |
| **Psychosocial factors** | | | | | | | | |
| Depression | Psychological distress (Kessler) | Screening | 10 | 5-*none of the time to all of the time* |  | 0.870 | Kessler-10 (28, 29) | Sum modeled dichotomous variable - > 16 vs. <16 |
| Disclosure | Disclosed | Baseline, Follow-ups 1 & 2 | 2 |  | *Have you told your partner that you are HIV+?*  Have you told anyone else? [**IF YES]**  Whom did you tell? |  |  | Dichotomous variable – disclosed to anyone vs. no one |
| Coping strategies | Coping strategies:  a. Acceptance  b. Alcohol  c. Positive reframing  d. Denial  e. Religion | Baseline | 2 items each, except 1 item for alcohol | 4-*have not been doing this at all to have been doing this most of the time* | 1. *You’ve been learning to live with it.* 2. *You’ve been using alcohol or other drugs to make yourself feel better* 3. *You’ve been looking for something good in what is happening.* 4. *You’ve been refusing to believe that it has happened.* 5. *You’ve been praying or reflecting* | 0.828  N/A  0.506  0.623  0.771 | Selected subscales from Brief Cope (Carver, 1997)(30) | Mean modeled as continuous variables |
| Satisfaction in care | Satisfaction in care | Baseline, Follow-ups 1 & 2 | 12 items | 4-*strongly disagree to strongly agree* | *When you visited the clinic to get tested for HIV, (the counsellor/nurse) who saw you….. Judged you for being HIV+.* | 0.803 | Developed during formative work | Mean modeled as a 3-level categorical variable (tertiles) |
| **Health Status Indicators** | | | | | | | | |
| HIV symptoms/disease severity | CD4+ count | Assessment when they linked to care | 1 |  | Only asked of those who returned to clinic and obtained CD4 count: *Can you tell me your CD4 count?* |  |  | 3-level categorical variable -  <150, 150-299,  >= 300 |
|  | WHO stage 3/4 clinical criteria | Screening |  |  | Self-reported - clinical criteria for WHO stage III/IV disease |  | World Health Organization (31) | Number of WHO stage 3/4 clinical signs or symptoms; dichotomous - none, one or more |

^a^**Note regarding timing of assessments:** The Screening Interview was conducted prior to diagnosis; the Post-Test Interview was conducted immediately after diagnosis; the Baseline Interview was conducted after diagnosis on the same day or within 30 days thereafter; Follow-up 1 and follow-up 2 were conducted approximately 4 and 8 months from diagnosis.
