# Supplementary material for: Predictors of antiretroviral therapy initiation in eThekwini (Durban), South Africa: Findings from a prospective cohort study
Source: PLoS One. 2021 Feb 19;16(2):e0246744. doi: 10.1371/journal.pone.0246744 (PMC7895397; doi:10.1371/journal.pone.0246744)
Supplement: S2 Table — (DOCX) [file pone.0246744.s003.docx]

| **Table 2**. **Hazard ratios (HR) for hypothesized predictors of time to ART initiation (adjusted for clinic and confounders) (N=153) stratified by gender** | | | | | |
| --- | --- | --- | --- | --- | --- |
|  | **ART Initiation** | | | |  |
|  | **Females (n=99)** | | **Men (n=54)** | |  |
|  | **HR** | **95% CI** | **HR** | **95% CI** |  |
| **Social structural factors** |  |  |  |  |  |
| Age (continuous)^a^ | **1.05** | **1.02-1.09** | 1.01 | 0.949-1.07 |  |
| Education^c^ (ref: 8th grade or less) |  |  |  |  |  |
| 9th - 11th grade | 1.45 | 0.79-2.67 | 1.98 | 0.58-6.80 |  |
| Grade 12- matriculated | 1.38 | 0.65-2.96 | 1.87 | 0.41-8.58 |  |
| Matriculation plus certificate, diploma, degree | 1.06 | 0.45-2.54 | 5.90 | 1.54-22.61 |  |
| Employed, yes^c^ (ref: no) | 1.23 | 0.73-2.08 | 0.71 | 0.34-1.46 |  |
| Food Insecurity, ever^c^ (ref: never) | 0.94 | 0.58-1.52 | 0.39 | 0.16-0.91 |  |
| Relationship status^b^ (ref: not married or in a relationship) |  |  |  |  |  |
| Married/in a relationship, not living together | 1.11 | 0.51-2.40 | 1.57 | 0.34-7.32 |  |
| Married/in a relationship, living together | 0.66 | 0.27-1.58 | 1.43 | 0.31-6.56 |  |
| Away from home, yes^b^ (ref: no) | 1.08 | 0.45-2.55 | 1.50 | 0.52-4.35 |  |
| Gender-related barriers^b^ (ref: 0) |  |  |  |  |  |
| 0.1 to 1 | 1.05 | 0.58-1.88 | 1.80 | 0.53-6.12 |  |
| 1.1 to 2 | 1.23 | 0.62-2.44 | 2.66 | 0.66-10.68 |  |
| Internalized stigma, any, less than 4 weeks after linkage to care ^b^ (ref: none) | 1.03 | 0.55-1.91 | 0.55 | 0.25-1.25 |  |
| Internalized stigma, any, greater than or equal to 4 weeks after linkage to care^b^ (ref: none) | 1.27 | 0.67-2.42 | 0.55 | 0.25-1.25 |  |
| Anticipated stigma, high^b^ (ref: low) | 1.04 | 0.57-1.90 | 0.55 | 0.22-1.36 |  |
| Shame, high^b^ (ref: low) | 0.74 | 0.43-1.27 | 0.89 | 0.36-2.22 |  |
| Blame, high^b^ (ref: low) | 0.90 | 0.52-1.56 | 0.41 | 0.17-1.00 |  |
| Travel time to clinic^d^ (ref: < ½ hour) |  |  |  |  |  |
| 1/2 hr.- 1 hr | 0.71 | 0.42-1.20 | 3.48 | 1.31-9.22 |  |
| > 1 hr. | 1.03 | 0.31-3.44 | 0.73 | 0.16-3.45 |  |
| GPS distance^d^ (ref: < 1.42 km) |  |  |  |  |  |
| 1.421 - 2.620 km | 0.67 | 0.37-1.22 | 1.06 | 0.46-2.48 |  |
| > 2.62 km | 0.93 | 0.52-1.68 | 1.41 | 0.55-3.59 |  |
| Cost for visit^d^ (ref: none) |  |  |  |  |  |
| Between R1.00-R15.00 | 0.91 | 0.54-1.55 | 1.35 | 0.57-3.16 |  |
| R15.00+ | 0.48 | 0.20-1.17 | 0.67 | 0.21-2.09 |  |
| **Social-cognitive factors** |  |  |  |  |  |
| CD4 knowledge^f^ (ref: none correct) |  |  |  |  |  |
| 1 correct | 0.64 | 0.26-1.54 | 1.39 | 0.40-4.88 |  |
| 2 correct | 0.96 | 0.46-2.01 | 0.93 | 0.33-2.65 |  |
| Negative outcome beliefs, any^e^ (ref: none) | 0.78 | 0.46-1.31 | 0.95 | 0.47-1.91 |  |
| Positive outcome beliefs, any^e^ (ref: none) | 1.11 | 0.57-2.13 | 2.33 | 0.67-8.15 |  |
| ARV positive attitudes^e^ (ref: 1^st^ tertile) |  |  |  |  |  |
| 2nd tertile | 1.34 | 0.76-2.38 | 0.98 | 0.10-9.74 |  |
| 3rd tertile | 0.65 | 0.32-1.32 | 1.27 | 0.55-2.93 |  |
| Stronger beliefs in traditional medicine^e^ (ref: 1^st^ tertile) |  |  |  |  |  |
| 2nd tertile | 1.46 | 0.73-2.92 | 0.75 | 0.31-1.84 |  |
| 3rd tertile | 0.73 | 0.39-1.39 | 0.38 | 0.17-0.89 |  |
| Care satisfaction^k^ (ref: 1^st^ tertile) |  |  |  |  |  |
| 2nd tertile | 0.85 | 0.48-1.52 | 0.93 | 0.36-2.41 |  |
| 3rd tertile | 0.98 | 0.53-1.80 | 0.77 | 0.36-1.65 |  |
| **Psychosocial factors** |  |  |  |  |  |
| Psychological distress (Kessler) sum, elevated > 16 ^h^ (ref: not elevated < 16) | 0.73 | 0.31-1.73 | ****** | ****** |  |
| Disclosed by baseline interview, yes^j^ (ref: no) | 0.93 | 0.55-1.60 | 1.06 | 0.47-2.35 |  |
| Coping acceptance^i^ (continuous) | 0.86 | 0.69-1.07 | 0.94 | 0.61-1.43 |  |
| Coping alcohol^i^ (continuous) | 1.80 | 0.84-3.83 | 0.52 | 0.22-1.19 |  |
| Coping positive reframing^i^ (continuous) | 0.75 | 0.55-1.02 | 1.11 | 0.72-1.69 |  |
| Coping denial^i^ (continuous) | 0.97 | 0.63-1.48 | 1.45 | 0.67-3.10 |  |
| Coping religion^i^ (continuous) | 0.99 | 0.79-1.23 | 1.19 | 0.72-1.95 |  |
| **Health status indicators** |  |  |  |  |  |
| CD4+ count^g^ (ref: <150 cells/μL) |  |  |  |  |  |
| 150-299 cells/μL | 0.71 | 0.43-1.18 | 0.97 | 0.44-2.12 |  |
| >= 300 cells/μL | 0.37 | 0.16-0.88 | 0.32 | 0.07-1.49 |  |
| WHO stage 3/4 clinical criteria, one or more^g^ (ref: none) | 0.67 | 0.40-1.11 | 0.54 | 0.27-1.08 |  |
| ^a-k^As indicated below, variables included in adjusted models are based on the Directed acyclic graph (S1 Table) | | | | | |
| ^a^Adjusted for clinic | | | | | |
| ^b^Adjusted for clinic, age, gender | | | | | |
| ^c^Adjusted for clinic, age, gender, relationship status | | | | | |
| ^d^Adjusted for clinic, education | | | | | |
| ^e^Adjusted for clinic, age, gender, psychological distress, CD4 knowledge, education | | | | | |
| ^f^Adjusted for clinic, age, gender, education | | | | | |
| ^g^Adjusted for clinic, age, gender, delayed testing, education | | | | | |
| ^h^Adjusted for clinic, age, gender, internalized stigma | | | | | |
| ^i^Adjusted for clinic, age, gender, psychological distress | | | | | |
| ^j^Adjusted for clinic, age, gender, psychological distress, gender barrier scale, anticipated stigma, ARV positive attitudes | | | | | |
| ^k^Adjusted for clinic, CD4 knowledge, traditional medicine attitudes | | | | | |
| Significant p-values (p<0.05) are highlighted in bold. | | | | | |
| *Positivity assumption violated (no event of ART initiation among the exposed group) | | | | | |
